# PROFILES IN FORTITUDE: STATEWIDE AGE-FRIENDLY HEALTH SYSTEMS

**DOI:** 10.1093/geroni/igae098.1044

**Published:** 2024-12-31

**Authors:** Teri Kennedy

**Affiliations:** University of Kansas Medical Center, Kansas City, Kansas, United States

## Abstract

The University of Kansas is in the process of developing Age-Friendly Kansas Forum (Kansas 4M), a statewide Kansas Age-Friendly Health Systems Action Community that is a reciprocal partnership between academia and primary care sites, delivery systems, and community organizations serving older adults in rural and underserved communities across Kansas. The Age-Friendly Health Systems (AFHS) Framework, developed by The John A. Hartford Foundation and the Institute for Healthcare Improvement, addresses the needs and priorities of older adults through the lens of the 4Ms: 1) what matters, 2) medications, 3) mobility, and 4) mentation. The framework resulted from a distillation of the core elements common to evidence-based programs serving older adults and is being leveraged as a strategy to address the know-do gap. To date three other states, Michigan, New York, and Nebraska, have developed statewide AFHS Action Communities. This presentation will share what Kansas 4M has learned about the unique approach taken by each of these states in their efforts to launch a statewide AFHS Action Community, their unique and shared experiences, best practices, cautionary tales, and how this information is informing the development of Kansas’ statewide AFHS Action Community.

